# Emotional Intelligence and Alcohol Consumption in the Tunisian Population

**DOI:** 10.1192/j.eurpsy.2026.10852

**Published:** 2026-07-31

**Authors:** I. Mannoubi, M. Turki, M. Ayedi, M. A. Megdiche, N. Halouani, S. Ellouze, J. Aloulou

**Affiliations:** Psychiatry B, Hedi Chaker hospital, Sfax, Tunisia

## Abstract

**Introduction:**

Alcohol consumption is a major public health issue influenced by multiple factors. Emotional intelligence (EI), defined as the ability to perceive, understand, and regulate one’s own and others’ emotions, may modulate drinking behavior by affecting stress management and emotional regulation.

**Objectives:**

To explore the relationship between alcohol consumption and EI.

**Methods:**

A descriptive and analytical cross-sectional observational study was conducted among 161 Tunisian participants using an anonymous online questionnaire (Google Forms). Two validated French tools were employed: the Alcohol Use Disorders Identification Test (AUDIT) for alcohol consumption and the Schutte Self-Report Emotional Intelligence Test (SSEIT) for EI.

**Results:**

Among alcohol consumers (N=63), the mean AUDIT score was 6.27 ± 5.7 (range: 1–28), with 15.9% meeting criteria for alcohol dependence. The mean SSEIT score was 115.52 ± 26.23 (range: 33–165), with 54% of participants showing moderate EI. A significant negative correlation was found between SSEIT and AUDIT scores (r=–0.19; p = 0.01), indicating that higher EI is associated with lower alcohol consumption. Alcohol dependence was linked to lower SSEIT scores, although this difference was not statistically significant.

**Conclusions:**

Lower EI may increase vulnerability to using alcohol as a maladaptive emotional regulation strategy, whereas higher EI supports more effective coping. These findings highlight the potential role of EI in preventive strategies and risk behavior identification.

**Disclosure of Interest:**

None Declared

